# Effects of ZnO nanoparticles concentration on the morphology and textural properties of ZnO/NiFe_2_O_4_ nanocomposite

**DOI:** 10.1016/j.mex.2025.103199

**Published:** 2025-02-03

**Authors:** Jimoh Oladejo Tijani, Augustine Innalegwu Daniel, Sarah Udenyi Onogwu, Ambali Saka Abdulkareem, Usman Ahmed Aminu, Marshall Keyster, Ashwil Klein

**Affiliations:** a Department of Chemistry, Federal University of Technology, PMB 65, Minna, Niger, Nigeria; b Department of Biochemistry, Federal University of Technology, PMB 65, Minna, Niger, Nigeria; c Department of Chemistry, Joseph Sarwuan Tarka University, PMB 2373, Makurdi, Benue, Nigeria; d Department of Chemical Engineering, Federal University of Technology, PMB 65, Minna, Niger, Nigeria; e Nanotechnology Research Group, Africa Centre of Excellence for Mycotoxin and Food Safety, Federal University of Technology, PMB 65, Bosso, Minna, Niger, Nigeria; f Department of Biotechnology, University of the Western Cape, Robert Sobukwe Road, Bellville, 7535, South Africa

**Keywords:** ZnO/NiFe_2_O_4_, Nanocomposites, Green synthesis, Co-precipitation, Sol-gel, EELS, XPS and BET, Green synthesis and Co-precipitation method

## Abstract

The aim of this study is to synthesize and characterize ZnO and NiFe_2_O_4_ nanoparticles via green route and co-precipitation of ZnO/NiFe_2_O_4_. X-ray diffraction (XRD) data show no extra diffraction peaks belonging to other phases except wurtzite. High resolution transmission electron microscopy (HRTEM) images showed that the average interplanar distance of wurtzite phase at 3, 5, and 7 % dopant concentration were about 0.28, 0.44 and 0.33 nm respectively. X-ray photoelectron spectroscopy (XPS) results show difference in binding energies of the elements present in different concentration of the dopants. Electron Energy Loss Spectroscopy (EELS) spectra show similarities in the shape of Zn, Fe and Ni from zero loss, low loss and core loss region with a little shift in energy. All the elements exhibit multiple oxidation state; +2 and +3 for Fe and +1 and +2 for Zn and Ni. Brunauer-Emmett-Teller (BET) plot shows that ZnO belongs to the type II isotherm curve while NiFe_2_O_4_ and 3, 5 and 7 % ZnO/NiFe_2_O_4_ all belong to type IV isotherm curve indicating ZnO as macroporous while NiFe_2_O_4_ and different dopant concentration of ZnO/NiFe_2_O_4_ are mesoporous. The study shows the complete synthesis of ternary ZnO/NiFe_2_O_4_ nanocomposites using green synthesis and sol-gel approach.

• Green synthesis of ZnO and NiFe_2_O_4_ using leaf extract of *Anacardium occidentale*
• Co-precipitation method at different concentration of ZnO and NiFe_2_O_4_ for the synthesis of ZnO/NiFe_2_O_4_.
• Nanocomposites was characterized using different analytical tools

## Graphical abstract

**Figure:**
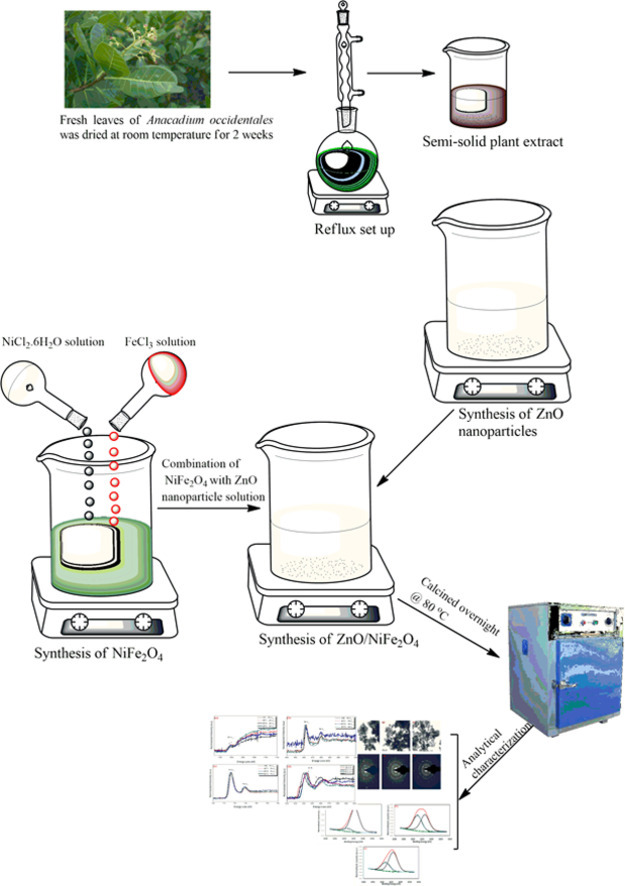

Specifications table

**Table:** 

Subject area:	Chemistry
More specific subject area:	Synthesis and characterization of nanocomposites
Name of your method:	Green synthesis and Co-precipitation method
Name and reference of original method:	NA
Resource availability:	The study was carried out in the Centre for Genetic Engineering and Biotechnology Laboratory of Nanotechnology Research Group, Africa Centre of Excellence for Mycotoxin and Food Safety, Federal University of Technology, PMB 65, Bosso, Minna, Niger State, Nigeria. The reagents and chemicals used were purchased from commercial suppliers such as Merck, Sigma-Aldrich. The instruments used were HRTEM, XRD, XPS, EELS and BET.

## Background

Zinc oxide nanoparticles, an n-type semiconductor with a 3.3 eV band gap, have attracted a lot of attention because of its great mechanical and thermal stability at ambient temperature, significant excitonic binding energy, oxidation and photonic resistance, and non-toxicity [1,2]. Because of their distinctive properties, ZnO nanoparticles find wide-ranging applications in electronic devices, astringents, laser technology, veterinary science, as well as in antigenic and antibacterial agents [3]. Zinc oxide (ZnO) nanoparticles can be produced by many chemical techniques, including hydrothermal synthesis [4], precipitation [5], chemical vapor transport [6], and sol-gel [7]. However, these conventional methods of synthesis have several drawbacks, such as energy and time-inefficient and the use of harmful chemicals as capping and stabilizing agents that endanger the environment [7]. Thus, green synthesis of nanoparticles which involves the use of biological materials such as plants, seaweeds, and microbes have better advantage than the chemical method because of they are safer, non-toxic, cost-effective, environmentally friendly, and bio-compatible [8,9]. ZnO nanoparticles are commonly employed as photocatalysts, yet their effectiveness is hindered by their wide band gap, which allows only 4 % of the solar spectrum to be utilized. To optimize and enhance their solar spectrum usage, ZnO nanoparticles can be doped with metals, non-metals, or metal oxides into a composites form. In this study, NiFe_2_O_4_ was used as a potential metal oxide for doping ZnO.

NiFe_2_O_4_ nanoparticles is a spinel ferrites nanoparticle that has garnered considerable interest in the scientific community, particularly in nanoscience, owing to its unique magnetic properties and wide-ranging applications in photocatalysis, microwave devices, magnetic nanocarriers for drug delivery and gas sensors [10]. These applications are attributed to their smaller average grain size, high surface area, magnetic nature and crystallinity [11]. NiFe_2_O_4_ nanoparticles exhibit soft magnetic characteristics and have been proposed for scientific applications in stealth anti-radar devices. The material's exceptional characteristics, including its high permeability, significant magnetization, and absence of electromagnetic properties, make it very suitable for high frequency applications. The magnetic characteristics of the material are contingent upon its particle size and shape [12]. The Jahn Teller ion, which is responsible for the electrical and magnetic characteristics of nickel ferrite, is another of its crucial features [13]. Due to its small size NiFe_2_O_4_ nanoparticles has high surface-to-volume ratio, consequently increasing the surface area for interactions with other materials to occur [10]. Tijani et al. [2] had earlier reported the adsorptive and photocatalytic performance of ZnO/NiFe_2_O_4_ nanocomposites, however the effect of mixing ratios of NiFe_2_O_4_ nanoparticles on the crystal layers of ZnO nanoparticles and the physic-chemical properties of the ZnO/NiFe_2_O_4_ nanocomposites especially using XPS and EELS were not explored.

Therefore, this study involves a two-step synthesis of ternary ZnO/NiFe_2_O_4_ nanocomposite. Firstly, ZnO and NiFe_2_O_4_ nanoparticles were synthesized using leaf extract of *Anacardium occidentale* and secondly, synthesis of ZnO/NiFe_2_O_4_ by sol-gel method. The nanocomposite was further characterized using different analytical techniques.

## Method details

### Materials

Iron (III) chloride hexahydrate (≥99.99 %), nickel (II) chloride hexahydrate (99.9 %), zinc acetate dihydrate (99.99 %), and sodium hydroxide (≥97.0 %) were all purchased from Sigma Aldrich and used as received.

### Preparation and extraction of plant extract

*Anacardium occidentale* leaves were harvested, cleansed, and air-dried for 2 weeks at room temperature. The dried leaves were pulverized into fine powder using a kitchen blender. Exactly 50 g of the powdered leaf sample was measured and extracted with 400 mL of double distilled water under reflux for 2 h at 40 °C. The extract filtered using Whatman No. 1 filter paper. The partially dried extract was weighed into a sterile container and stored at 4 °C [14]. The green synthesis of ZnO and NiFe_2_O_4_ nanoparticles have been reported earlier by Tijani et al. [2].

### Co-Precipitation synthesis of ZnO/NiFe_2_O_4_

ZnO/NiFe_2_O_4_ nanocomposites was synthesized by co-precipitation method, following the protocol outlined by He et al. [15]. Exactly 1.0 g of FeCl_3_·6H_2_O and 0.56 g of NiCl_2_·6H_2_O were combined and dissolved in 40 mL of distilled water, resulting in a brown-green solution. 3 % (w/v) of ZnO nanoparticles in double distilled water were ultrasonicated for 60 min. Afterwards, the two solutions were combined, and the pH of the resulting solution was adjusted to 10 by gradually adding drops of a 2 M NaOH solution. The solution was agitated for another 1 h before centrifuging at 3000 rpm for 30 min to collect the nanomaterials. The nanomaterial was thoroughly washed with double distilled water and ethanol to eliminate any remaining contaminants, and then dried in the oven overnight at 80 °C. The dried sample was calcined at 600 °C to obtain crystalline ZnO/NiFe_2_O_4_ nanoparticles. The above procedure was repeated for 2.5 g and 1.56 g of ZnO to obtain 5 and 7 % dopant concentration [15]. The percentage of the dopant in each case was calculated using Eq. (1):

(1) $$\frac{m\left(\text{ZnO}\right)}{m\left(ZnO/\text{NiF}e_{2}O_{4}\right)}\times \frac{100}{1}=\varepsilon \%$$

Where m(NiFe_2_O_4_) is the mass of Nickel Ferrite added, m(ZnO) is the mass of ZnO nanoparticles.

The stoichiometric reaction between FeCl_3_, NiCl_2_, and NaOH to form nickel ferrite (NiFe_2_O_4_) is represented in Eq. 2 below:

(2) 2FeCl_3_ + NiCl_2_ + 8NaOH→NiFe_2_O_4_ + 8NaCl + 4H_2_O

### Characterization

The crystal structure of the sample was determined using a Bruker 8-Advance X-ray diffractometer. X-ray photoelectron spectroscopy (XPS) (TFSK-250XI) was used to measure the XPS spectra of the samples. The morphology of the materials was analyzed using Zeiss Auriga high resolution transmission electron microscopy combined with selected area electron diffraction (HRTEM-SAED). Electron Energy-Loss Spectroscopy (EELS) FEI Titan80-300 was used to determine the chemical composition, energy loss and electronic structure of the nanoparticles.

## Method validation

### XRD analysis

**Fig. 1:**
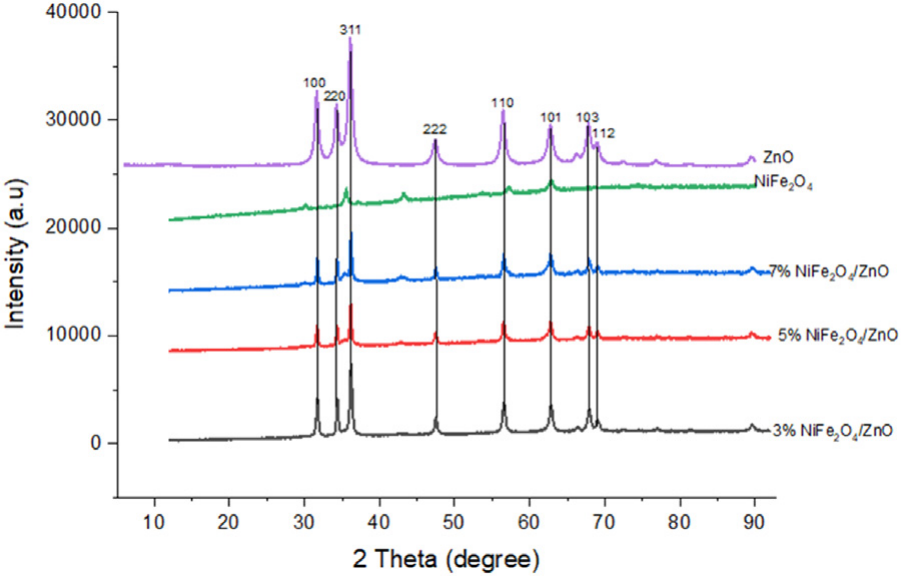
XRD pattern of magnetic ZnO/NiFe_2_O_4_ nanocomposites.

The phase of the synthesized ZnO/NiFe_2_O_4_ nanocomposites was determined using XRD analysis and the result is presented in Fig. 1. The nanocomposites at the different dopant concentrations shows a diffraction peaks at 2*θ* values of 30.32°, 35.34°, 37.21°, 47.23°, 56.71°, 63.40°, and 67.51° with the following crystal plane (100), (220), (102), (311), (222), (110) and (103) which corresponds to hexagonal wurtzite phase of ZnO. No extra diffraction peaks belonging to the other phases were observed suggesting that the synthesized spinel phase of NiFe_2_O_4_ is pure and the intensity of ZnO peaks gradually increases with increasing NiFe_2_O_4_ content. Moreover, the outcome also demonstrates the effective spreading of Fe_2_O_4_ over the pores of ZnO without any alteration or damage to the ZnO phase. The mean grain size of the ZnO/NiFe_2_O_4_ nanocomposites synthesized with dopant concentrations of 3, 5, and 7 % were determined to be 21.39 nm, 28.03 nm, and 36.18 nm, respectively. The correlation between the quantity of NiFe_2_O_4_ nanoparticles and the enlargement of grain size is evident. In addition, it was noted that the strength of the most prominent peaks with miller indices (100), (311), and (110) reduced, but the full width at half maximum (FWHM) increased as the number of NiFe2O4 nanoparticles increased from 3 to 7 %.

### HRTEM-SAED analysis

**Fig. 2:**
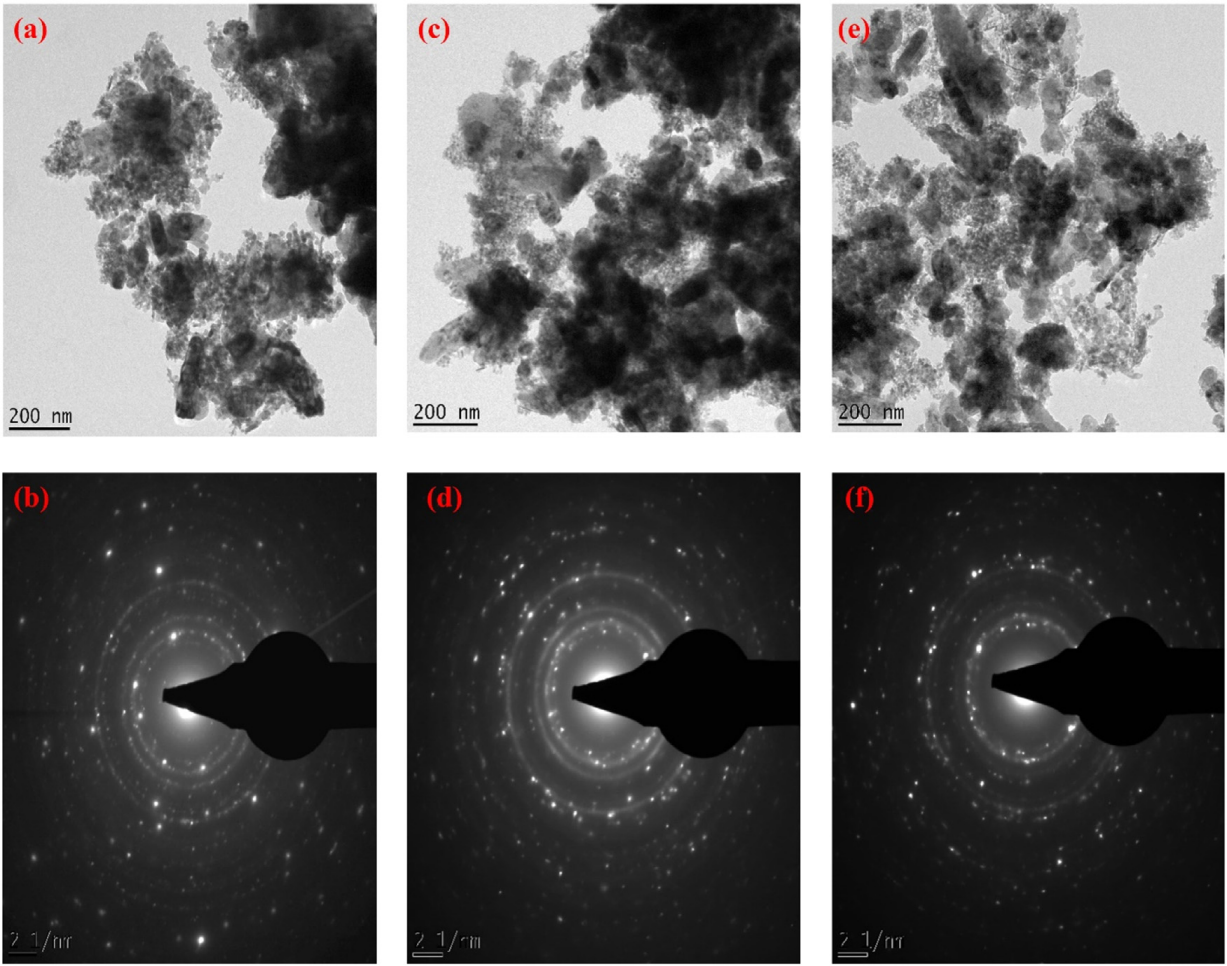
HRTEM and SAED micrograph of (a and b) 3 % (c and d) 5 % and (e and f) 7 % of ZnO/NiFe_2_O_4_ nanocomposites respectively.

Synthesis and characterization of ternary ZnO/NiFe_2_O_4_ nanocomposites was carried out using leaf extract of *A. occidentale.* The results of HRTEM and SAED micrographs of 3, 5 and 7 % ZnO/NiFe_2_O_4_ nanocomposites are presented in Fig. 2a–f. The nanostructures exhibited a spherical morphology, with minimal clustering (Fig. 2a). However, as the concentration of the dopant increased to 5 and 7 %, there was a complete spherical shape formation which is an indication of complete formation of ZnO/NiFe_2_O_4_ nanocomposites. The shape formation is due to the overlap between NiFe_2_O_4_ and ZnO along their direction of growth [16]. The arrangement of the lattice fringes of about 0.37, 0.33 and 0.31 nm were obtained for 3, 5 and 7 % nanocomposites respectively which corresponds to (311), (110), (101) crystal planes (Fig. 2b, d and f). This observation provides more evidence for the increasing alignment of the nanocomposites in the ZnO wurtzite structure (Fig. 2b, d and f).

### HRSEM analysis

**Fig. 3:**
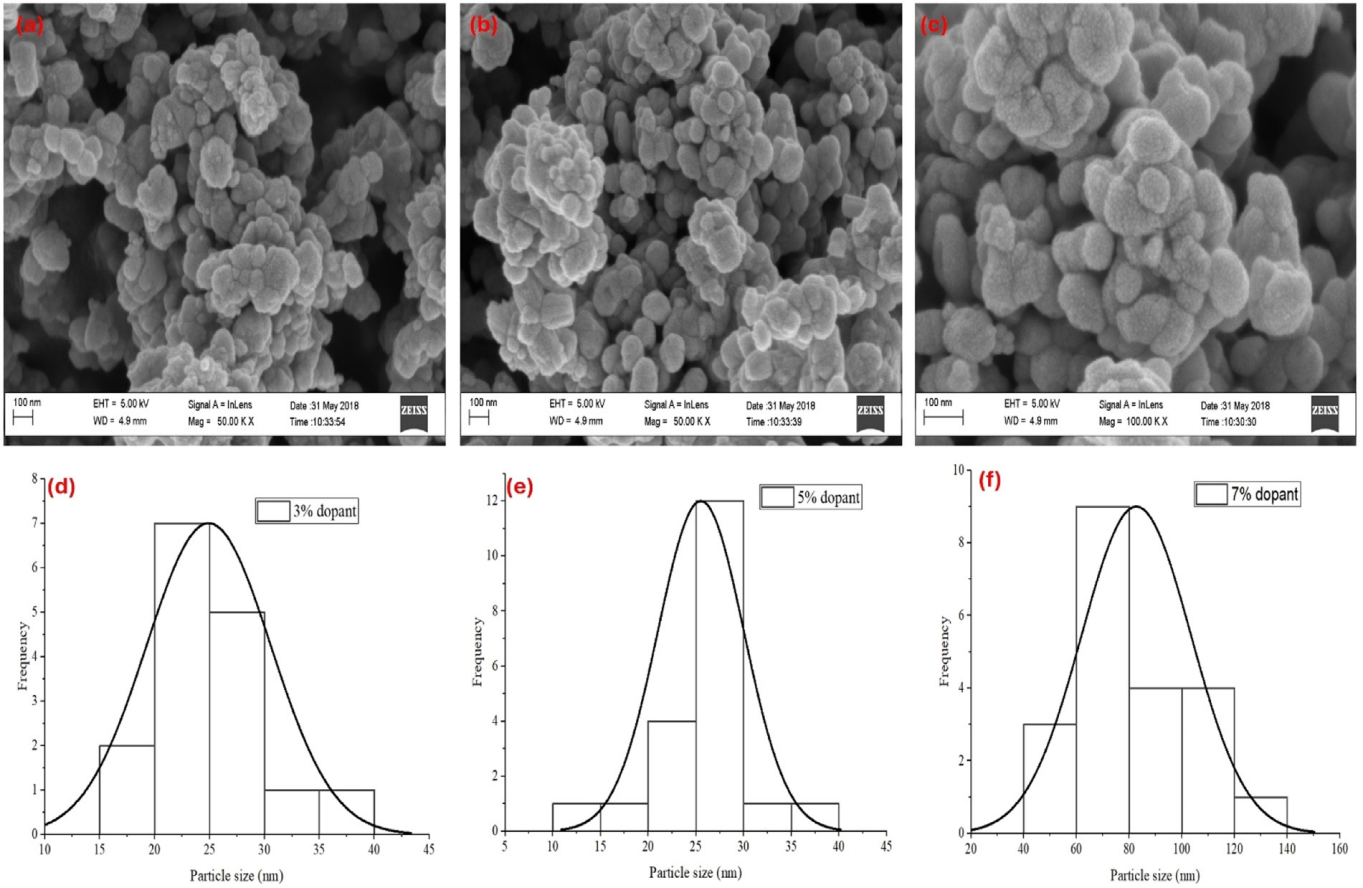
HRSEM and Particle size distribution analysis of (a and d) 3 % (b and e) 5 % and (c and f) 7 % of ZnO/NiFe_2_O_4_ nanocomposites respectively.

Fig. 3a–c shows HRSEM micrographs of the ZnO/NiFe_2_O_4_ nanocomposites produced using different concentration of the dopant. The micrograph indicates minimal morphological changes with increasing concentration of the dopant characterized by nano-scale pores surrounded by dense agglomerated regions (Fig. 3a–c). Furthermore, there was a significant increase in particle sizes of the nanocomposites from 24.91 nm at 3 % dopant concentration to 25.51 nm (5 % dopant) and 82.71 nm at 7 % dopant concentration (Fig. 3d–f). The observed increase in particle size of ZnO/NiFe₂O₄ nanocomposites with increasing ZnO concentration (3 %: 24.91 nm, 5 %: 25.52 nm, and 7 %: 82.72 nm) can be attributed to the growth and agglomeration tendencies of ZnO nanoparticles. At lower concentrations (3 % and 5 %), ZnO particles are more evenly distributed within the NiFe₂O₄ matrix, minimizing growth due to spatial restrictions. However, at higher ZnO concentration (7 %), the excess ZnO facilitates enhanced nucleation and coalescence of particles, resulting in larger ZnO domains [17,18]. This increased particle size can also be influenced by reduced interfacial interactions between ZnO and NiFe₂O₄, leading to weaker confinement effects as ZnO dominates the nanocomposite structure [19,20]. These chemical interactions and aggregation effects highlight the concentration-dependent growth behavior of ZnO in the composite.

### XPS analysis

**Fig. 4:**
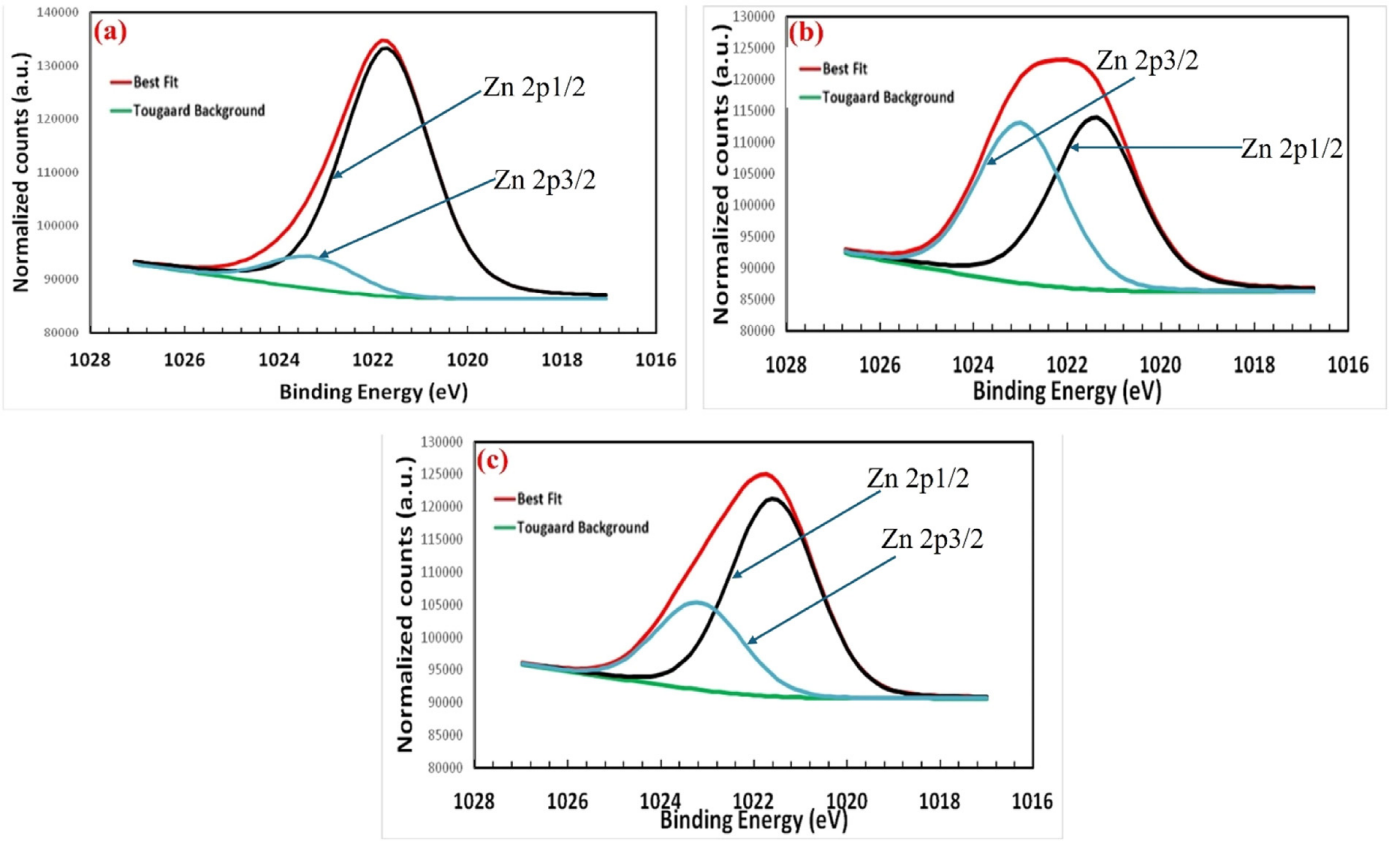
High resolution XPS spectrum of (a) 3 %, (b) 5 % and (c) 7 % of ZnO/NiFe_2_O_4_ nanocomposites.

X-ray Photoelectron Spectroscopy (XPS) is a powerful surface analysis technique that provides detailed information on the elemental composition, chemical state, and electronic structure of a material's surface. The surface oxidation state of the elements present in ZnO/NiFe_2_O_4_ nanocomposites were examined using XPS analysis (Fig. 4a–c).The results show high resolution XPS spectra for Zn (2p), Fe (2p), Ni (2p) in 3,5 and 7 % ZnO/NiFe_2_O_4._The binding energies of Zn (2p), Fe (2p), Ni (2p), O (1s) and C (1s) components of 3 % ZnO/NiFe_2_O_4_ (Fig. 4a) were significantly higher than the binding energies of Zn (2p), Fe (2p), Ni (2p), O (1s) and C (1s) components for 5 and 7 % of the dopant to form ZnO/NiFe_2_O_4_ nanocomposites (Fig. 4b and c). The difference in the binding energy may be due to 3d electron configurations of Fe, Ni, Zn, the spin–orbit coupling and electron–electron interactions within Fe, Ni, Zn and the hybridization of 3d electrons to other valence electrons [21]. Zn in the composites has 2p peaks at 1021.7 eV, which could be ascribed to the formation of zinc ferrite with zinc atom occupying tetrahedral sites. The peak at 1023.2 may be ascribed to Zn atom at the octahedral sites attributed to Zn 2p ($\frac{1}{2})$ and Zn 2p ($\frac{3}{2})$ of 3 % ZnO/NiFe_2_O_4_ nanocomposites respectively (Fig. 4a). Similarly, two fitting peaks located at 1042.6 and 1021.0 eV were attributed to Zn 2p ($\frac{1}{2})$ and Zn 2p ($\frac{3}{2})$ were observed for 5 % ZnO/NiFe_2_O_4_ nanocomposites respectively (Fig. 4b). However, Zn 2p core-level of 7 % ZnO/NiFe_2_O_4_ nanocomposites has two fitting peaks shifted towards the higher binding energy at 1043.2 and 1024.2 eV belonging to Zn 2p ($\frac{1}{2})$ and Zn 2p ($\frac{3}{2})$ orbital respectively (Fig. 4d). The Fe (2p) orbital in the nanocomposites occurred at binding energies of 725.5 eV and 709.4 eV and assigned as Fe 2p ($\frac{1}{2})$ and Fe 2p ($\frac{3}{2})$ for 3 % ZnO/NiFe_2_O_4_ nanocomposites (Fig. 4a). Also, in 5 % ZnO/NiFe_2_O_4_ nanocomposites the appearance of two peaks belonging to different binding energies of 716.4 eV and 710.7 eV was assigned to Fe 2p ($\frac{1}{2})$ and Fe 2p ($\frac{3}{2})$ respectively (Fig. 4b). In 7 % ZnO/NiFe_2_O_4_ nanocomposites, the 2p peaks located at 721.3 eV and 712.2 eV were ascribed to Fe 2p ($\frac{1}{2})$ and Fe 2p ($\frac{3}{2})$ respectively (Fig. 4c). Despite the different mixing ratio, Fe (2p) orbital remains constant, however, the values of the binding energies differ suggesting partial replacement or substitution of O in ZnO by Fe or Ni. Furthermore, the binding energy values of Ni 2p ($\frac{1}{2})$ and Ni 2p ($\frac{3}{2})$ peaks were 855.6 and 864.7 eV for 3 % nanocomposites (Fig. 4a), 867.0 and 854.5 eV for 5 % nanocomposites (Fig. 4b) 856.0 and 861.4 eV for 7 % ZnO/NiFe_2_O_4_ nanocomposites (Fig. 4c).

### EELS analysis

**Fig. 5:**
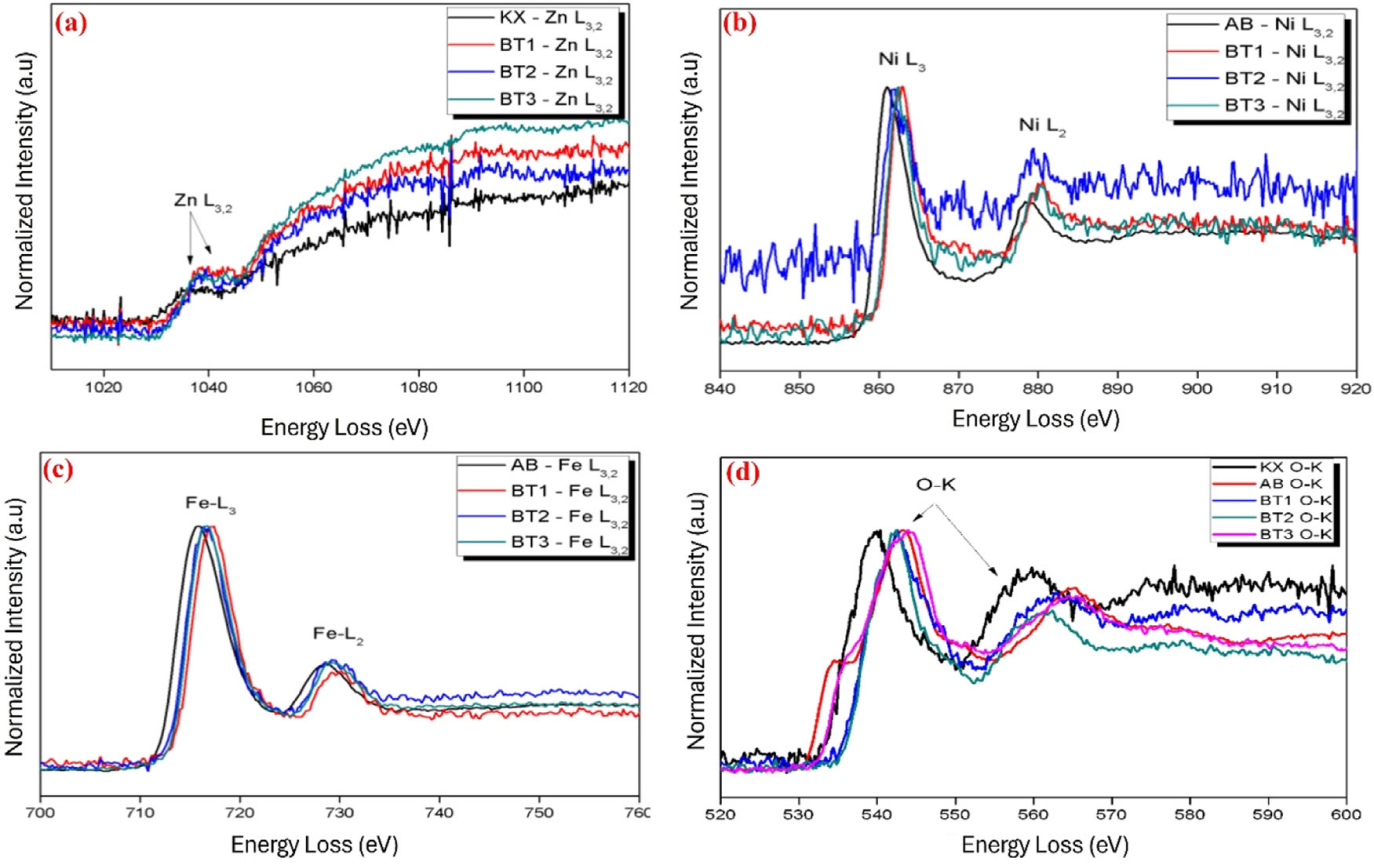
EELS spectrum of (a) Zn, (b) Ni, (c) Fe and (d) O in 3, 5 and 7 % ZnO/NiFe_2_O_4_ nanocomposites.

Chemical composition, energy loss and electronic structure of the synthesized ZnO/NiFe_2_O_4_ nanocomposites was determined using electron energy loss spectroscopy (Fig. 5a–d). The energy loss spectrum was obtained by quantifying the number of electrons that were lost at specific energy levels and graphing this data against the corresponding energy loss values. The spectra were subjected to critical analysis, which revealed the specific form of the Zn (L_2_ and L_3_), Fe (L_2_ and L_3_) and Ni (L_2_ and L_3_) edges and O (K) edge were similar in the three spectra from zero, low and core loss region with little shift in energy (Fig. 5a–d). These edges were conveniently classified according to the initial state of the excited electron. The shift in energy position and intensity ratio were found to mostly depend on the oxidation state of each element in the composite. It was observed that Zn and Ni in L_2_ and L_3_ orbitals exist in two different oxidation states of +1 and +2 respectively (Fig. 5a and b) while Fe exists in +2 and +3 oxidation state in L_2_ and L_3_ orbitals respectively (Fig. 5c). The slight shift in energy of the nanocomposites from zero to low loss region may be attributed to the delocalization effect of the weakly bonded outer shell electrons of Ni and not Fe. The delocalized electrons correlated with one another via electrostatic interactions. Moreover, the change in energy could potentially be associated with a significant presence of oxygen vacancies and the specific arrangement of atoms in the nanomaterial [22]. The increased presence of oxygen vacancies might be attributed to the diffusion of Fe and the coupling or partial substitution of Ni on the lattice layer of ZnO. The energy loss in the nanomaterials maybe attributed to the structural and electronic properties of nanomaterials [[23], [24], [25]]. This may arise from the intrinsic quantum confinement effects and electron density variations within the nanomaterials [[23], [25], [26], [27]]. The EELS analysis revealed that the nanomaterials possessed a crystalline structure. Furthermore, the inclusion of NiFe_2_O_4_ nanoparticles did not cause any changes to the wurtzite structure of the ZnO nanoparticles. The measured stoichiometric composition matched EDX report by [22]. In the region of increased energy loss, ionization of the Oxygen K-edge occurred in the L2 and L3 orbitals for Zn, Ni, and Fe. The simulation demonstrated that the initial characteristic in the oxygen K edge of ZnO/NiFe_2_O_4_ nanocomposites, with loadings of 3 %, 5 %, and 7 % NiFe2O4, occurred at approximately 543–561 eV. The nanomaterial exhibits potential transitions to the ultimate states (plasmon loss) through the vacant levels of Zn, Ni, and Fe [28].

### BET analysis

**Fig. 6:**
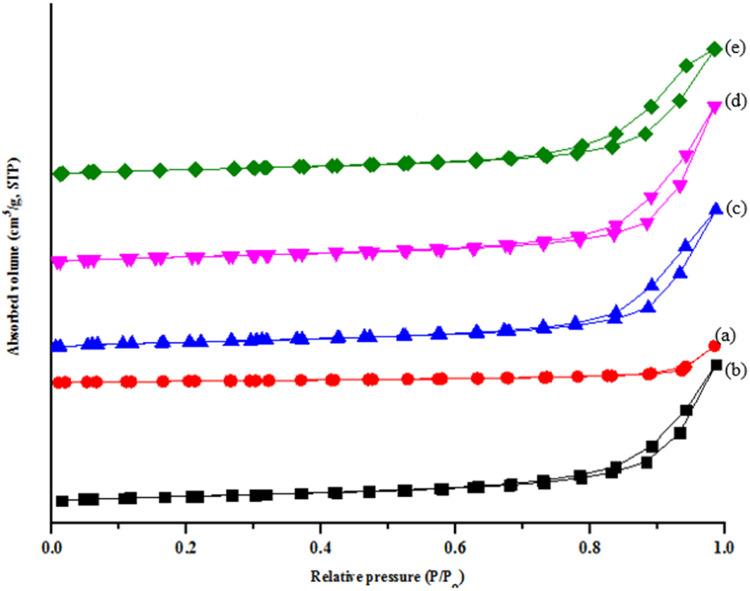
Nitrogen adsorption–desorption isotherms of (a) NiFe_2_O_4,_ (b) ZnO, (c) 7 % ZnO/NiFe_2_O_4_, (d) 5 % ZnO/NiFe_2_O_4,_ (e) 3 % ZnO/NiFe_2_O_4_ nanomaterials.

**Fig. 7:**
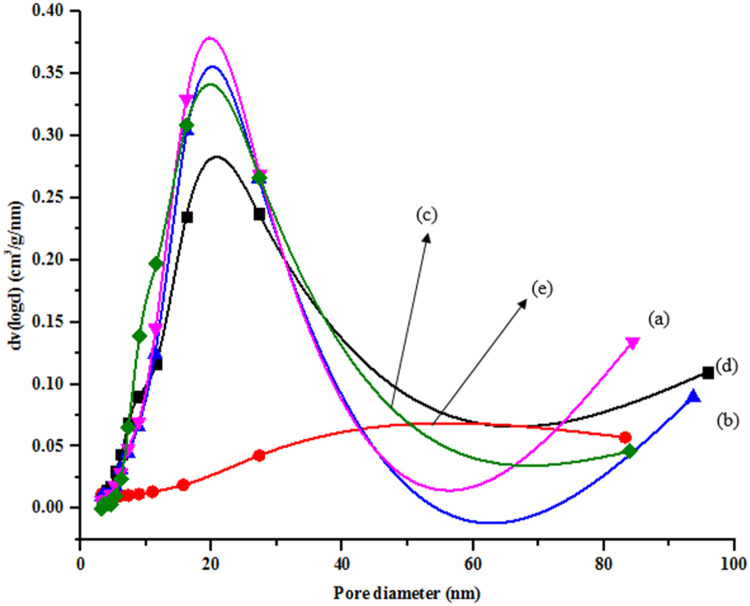
Pore size distribution curves of (a) 7 % ZnO/NiFe_2_O_4_, (b) 5 % ZnO/NiFe_2_O_4_, (c) 3 % ZnO/NiFe_2_O_4_, (d) NiFe_2_O_4,_ and (e) ZnO nanomaterials.

The surface area, pore volume, and pore size of ZnO/NiFe_2_O_4_ nanocomposites were determined using multiple-plot BET analysis (Table 1). The adsorption–desorption plot and pore size distribution of the nanomaterials is presented in Figs. 6 and 7. The N_2_ adsorption–desorption isotherms were employed to examine the BET specific surface area and porous structure of nanomaterials. NiFe_2_O_4_ and 7 % ZnO/NiFe_2_O_4_ nanomaterials has the least surface area (40.387 and 40.799 m^2^/g) and pore size (16.099 and 9.184 nm) respectively while 3 % ZnO/NiFe_2_O_4_ nanocomposites have the highest surface area of 44.693 m^2^/g with a surface area of 8.113 nm (Table 1). Based on IUPAC classification, the isotherms of the nanocomposites containing 3, 5, and 7 % NiFe_2_O_4_ fall under Type IV, characterized by a narrow hysteresis loop. These results agrees with the report of [29] who reported that both NiFe_2_O_4_ and ZnO/NiFe_2_O_4_ nanocomposites exhibited mesoporous characteristics. The isotherm of ZnO was classified as type II, which is indicative of the presence of a monolayer and macro porous structure in ZnO [29]. The increase in the surface area of ZnO nanoparticles, particularly when combined with NiFe_2_O_4_ in equal proportion, indicates the presence of a synergistic impact between these metals. Furthermore, increase in the amount of NiFe_2_O_4_ shows a significant increase in the surface area (Table 1). This was attributed to the excessive amount of Fe than Ni based on the ionic radii mechanism. The narrow range of pore size and decreased pore diameter in ZnO/NiFe_2_O_4_ nanocomposites can be attributed to the creation of a heterojunction and the coupling effect between NiFe_2_O_4_ and ZnO nanoparticles in the ZnO/NiFe_2_O_4_ nanomaterials [29]. The coupling effect resulted in the formation of an alloy (ZnO/NiFe_2_O_4_) and more active sites. The findings align with the report by [29], who observed an increase in the surface area of ZnO/NiFe_2_O_4_ nanoparticles produced using hydrothermal synthesis, from 4.73 to 59.89 m^2^/g for ZnO/NiFe_2_O_4_. The increase in the surface area was because of the incorporation of NiFe_2_O_4_ nanoparticles. Nevertheless, the surface area observed in this study is smaller than those reported by [29]. This disparity can be due to variations in the synthesis technique, types of precursor salts used, and the occurrence of doping phenomena. In addition, Xu et al. [30] observed an increase in the surface area of TiO_2_/NiFe_2_O_4_, which was synthesized using the sol-gel method, when TiO_2_ was combined with NiFe_2_O_4_. They ascribed the increase in the surface area to the characteristics of the precursor salt employed in the synthesis, the type of dopants, and the synthesis conditions.

**Table 1:** Surface area, pore volume and pore size distribution of the synthesized nanomaterials.

S/No.	Nanomaterial	Surface area (m^2^/g)	Pore vol. (cc/g)	Pore size (nm)
1	ZnO	8.988	0.353	13.173
2	NiFe_2_O_4_	40.387	0.214	16.099
3	ZnO/ NiFe_2_O_4_ (30/70)	44.693	0.190	8.113
4	ZnO/ NiFe_2_O_4_ (50/50)	43.707	0.202	11.091
5	ZnO/ NiFe_2_O_4_ (70/30)	40.799	0.204	9.184

## Conclusion

The synthesis of ZnO/NiFe_2_O_4_ using leaf extracts of *A. occidentale* as capping and reducing agent and was characterized using various analytical techniques. The surface area of ZnO slightly reduced with increasing concentration of NiFe_2_O_4_ into the lattice layer of ZnO as revealed by BET result. The shift in energy position and intensity ratio were found to mostly depend on the oxidation state of each element in the composite. Also, the differences in binding energies of the elements were attributed to the electron transition of the various metals (Fe, Ni and Zn) from their 3d orbitals to the empty 4s orbital during the ejection of the core 2p electrons as revealed by the XPS result. The chemical oxidation state of the respective elements was found to be +2 and +3 for Fe and +1 and +2 for Zn and Ni.

## Limitations

Not applicable.

## Ethics statements

Not applicable.

## CRediT authorship contribution statement

**Jimoh Oladejo Tijani:** Conceptualization, Methodology, Software, Data curation, Writing – original draft, Visualization, Investigation, Supervision, Validation, Writing – review & editing. **Augustine Innalegwu Daniel:** Conceptualization, Methodology, Software, Data curation, Writing – original draft, Visualization, Investigation, Supervision, Validation, Writing – review & editing. **Sarah Udenyi Onogwu:** Conceptualization, Methodology, Software, Writing – review & editing. **Ambali Saka Abdulkareem:** Conceptualization, Methodology, Software, Writing – review & editing. **Usman Ahmed Aminu:** Data curation, Writing – original draft, Visualization, Investigation, Supervision, Writing – review & editing. **Marshall Keyster:** Data curation, Writing – original draft, Visualization, Investigation, Supervision, Writing – review & editing. **Ashwil Klein:** Data curation, Writing – original draft, Visualization, Investigation, Supervision, Writing – review & editing.

## Declaration of competing interest

The authors declare that they have no known competing financial interests or personal relationships that could have appeared to influence the work reported in this paper.

## Data Availability

Data will be made available on request.

## References

[1] Adeleke J., Theivasanthi T., Thiruppathi M., Swaminathan M., Akomolafe T., Alabi A. (2018). Photocatalytic degradation of methylene blue by ZnO/NiFe_2_O_4_ nanoparticles. Appl. Surf. Sci..

[2] Tijani J.O., Aminu U., Bankole M.T., Ndamitso M., Abdulkareem A. (2020). Adsorptive and photocatalytic properties of green synthesized ZnO and ZnO/NiFe_2_O_4_ nanocomposites for tannery wastewater treatment. Niger. J. Technol. Dev..

[3] Raha S., Ahmaruzzaman M. (2022). ZnO nanostructured materials and their potential applications: progress, challenges and perspectives. Nanoscale Adv..

[4] Khudiar S.S., Mutlak F.A.H., Nayef U.M. (2021). Synthesis of ZnO nanostructures by hydrothermal method deposited on porous silicon for photo-conversion application. Optik.

[5] Ghorbani H.R., Mehr F.P., Pazoki H., Rahmani B.M. (2015). Synthesis of ZnO nanoparticles by precipitation method. Orient. J. Chem.

[6] Khan I., Rasheed A., Farid A., Raza A., Yousaf M., Abbas A. (2023). Structural, optical and dielectric properties of chemical vapor transport based synthesis of rice-like nanostructured cadmium zinc oxide films. Thin Solid Films.

[7] Yusha'u A., Darma M.S., Isah K.A. (2023). Sol-gel synthesis of ZnO nanoparticles for optmized photocatalytic degradation of eriochrome Black T under UV irradiation. Alger. J. Eng. Technol..

[8] Ismail S.M.M., Ahmed S.M., Abdulrahman A.F., AlMessiere M.A. (2023). Characterization of green synthesized of ZnO nanoparticles by using pinus brutia leaves extracts. J. Mol. Struct..

[9] Kavitha A., Doss A., Pole R.P., Rani T.K.P., Prasad R., Satheesh S. (2023). A mini review on plant-mediated zinc oxide nanoparticles and their antibacterial potency. Biocatal. Agric. Biotechnol..

[10] Manohar A., Vijayakanth V., Vinodhini V., Chintagumpala K., Manivasagan P., Jang E., Kim K. (2023). ESR, magnetic hyperthermia and cytotoxicity studies of Zn-doped NiFe_2_O_4_ nanoparticles. J. Alloy. Compd..

[11] Munir S., Warsi M.F., Zulfiqar S., Ayman I., Haider S., Alsafari I.A., Shakir I. (2021). Nickel ferrite/zinc oxide nanocomposite: investigating the photocatalytic and antibacterial properties. Journal of Saudi Chemical Society.

[12] Hemeda O.M., Mostafa N.Y., AbdElkader O.H., Hemeda D.M., Tawfik M., Mostafa M. (2015). Electrical and morphological properties of magnetocaloric nano ZnNi ferrite. Journal of Magnetism and Magnetic Materials.

[13] Wu X., Wu W., Qin L., Wang K., Ou S., Zhou K., Fan Y. (2015). Structure and magnetic properties evolution of nickel–zinc ferrite with lanthanum substitution. Journal of Magnetism and Magnetic Materials.

[14] Ogbadoyi E.O., Abdulganiy A.O., Adama T.Z., Okogun J.I. (2007). *In vivo* trypanocidal activity of Annona senegalensis Pers. Leaf extract against Trypanosoma brucei brucei. J. Ethnopharmacol..

[15] He Z., Xia Y., Tang B., Su J., Jiang X. (2019). Optimal co-catalytic effect of NiFe_2_O_4_/ZnO nanocomposites toward enhanced photodegradation for dye MB. Z. Phys. Chem..

[16] Moradi S., Taghavi Fardood S., Ramazani A. (2018). Green synthesis and characterization of magnetic NiFe_2_O_4_@ ZnO nanocomposite and its application for photocatalytic degradation of organic dyes. J. Mater. Sci. Mater. Electron..

[17] Wang Y., Gao X., Zhou H., Wu X., Zhang W., Wang Q., Luo C. (2019). Fabrication of biomass-derived carbon decorated with NiFe_2_O_4_ particles for broadband and strong microwave absorption. Powder Technol..

[18] Yu Y., Bei H., Huang H., Wu L., Zhao Y., Yin G., Pang H. (2024). Enhanced microwave absorption of coral-like Co@ Co_7_Fe_3_ at ultralow filler loading. J. Mater. Chem. C.

[19] Cheng G., Pan F., Zhu X., Dong Y., Cai L., Lu W. (2021). Onion skin-derived hierarchical carbon/hollow CoFe_2_O_4_ composite with effective microwave absorption in multi-band. Compos. Commun..

[20] Guan J., Li H., Ren J., Qiu W., Li Q., He Z., Zhu M., Li W., Jia N., Lu S. (2024). MOFs-derived flower-like cobalt@ carbon multiscale hierarchical composites with effective microwave absorption in the low frequency range. Int. J. Miner. Metall. Mater..

[21] Aghavnian T., Moussy J.B., Stanescu D., Belkhou R., Jedrecy N., Magnan H., Ohresser P., Arrio M.A., Sainctavit P., Barbier A. (2015). Determination of the cation site distribution of the spinel in multiferroic CoFe_2_O_4_/BaTiO_3_ layers by X-ray photoelectron spectroscopy. J. Electron Spectrosc. Relat. Phenom..

[22] Ding Y., Wang Z.L. (2005). Electron energy-loss spectroscopy study of ZnO nanobelts. J. Electron Microsc..

[23] Abass N.A.H.A., Jawad M.F., Haider A.J., Taha B.A. (2024). Exploring random laser characteristics in core@ shell nano-scatter centers: trends and opportunities. Opt. Quantum. Electron..

[24] Hamed A.S., Ali I.A., El Ghazaly M., Hassan H.E., Al-Abyad M. (2021). Multifunctional radioactive ZnO/NiFe2O4 nanocomposite for theranostic applications. The European Physical Journal Plus.

[25] Sahu A., Kumar D. (2022). Core-shell quantum dots: a review on classification, materials, application, and theoretical modeling. J. Alloy. Compd..

[26] Hamed A.S., Ali I., El Ghazaly M., Al-Abyad M., Hassan H. (2021). Nanocomposites of ZnO mixed with different Ni-ferrite contents: structural and magnetic properties. Phys. B Condens. Matter.

[27] R. Schneider. (2011). Electron energy-loss spectroscopy (EELS) and energy-filtering transmission Electron microscopy (EFTEM). Surface and Thin Film Analysis: A Compendium of Principles, Instrumentation, and Applications, 67–91.

[28] Krishna M.B.M., Madéo J., Urquizo J.P., Zhu X., Vinod S., Tiwary C.S., Ajayan P.M., Dani K.M. (2018). Terahertz photoconductivity and photocarrier dynamics in few-layer hBN/WS2 van der Waals heterostructure laminates. Semicond. Sci. Technol..

[29] Zhu H.Y., Jiang R., Fu Y.Q., Li R.R., Yao J., Jiang S.T. (2016). Novel multifunctional NiFe_2_O_4_/ZnO hybrids for dye removal by adsorption, photocatalysis and magnetic separation. Appl. Surf. Sci..

[30] Xu S.H., Tan D.D., Bi D.F., Shi P.H., Lu W., Shangguan W.F., Ma C.Y (2013). Effect of magnetic carrier NiFe_2_O_4_ nanoparticles on physicochemical and catalytic properties of magnetically separable photocatalyst TiO_2_/NiFe_2_O_4_. Chem. Res. Chin. Univ..

